# Socially engaged art practice and working equine welfare: creative methodologies for evolving how we see and attend to human-equine relationships

**DOI:** 10.3389/fvets.2026.1905865

**Published:** 2026-10-13

**Authors:** Glen Cousquer, Eszter Erdosi

**Affiliations:** 1 Royal (Dick) School of Veterinary Studies, University of Edinburgh, Edinburgh, United Kingdom; 2 Independent Researcher, Edinburgh, United Kingdom

**Keywords:** attention and awareness, oppression and exploitation, photographic practice, relational care practices, socially engaged arts practice, tethering, working equine welfare

## Abstract

Socially engaged art is a recognized field of practice in qualitative research and is increasingly being applied to addressing the complex health challenges we face in today's world. Its use has received minimal attention in the animal welfare sciences, however, despite being well suited to exploring, understanding, representing, and challenging human experiences, behaviors, assumptions, practices and cultures. Where exploitation of the more-than-human world is concerned, socially engaged practices can disrupt systems of oppression and stimulate transformational learning. This has particular relevance for relationships between equines and their humans. Art that is socially engaged can co-create new knowledges, helping us to reshape our understanding of relational care practices. As recent research has shown, a shift from animal labor to multispecies care can thus be developed, as art practices enable viewers to reacquaint themselves with taken-for-granted contexts of animal work habits that have become normative. This paper reviews the literature on socially engaged practice for human-mule welfare, before presenting an analysis of the work of a photographer and an artist who collaborated on the development of guidelines for pack animal welfare in Morocco's Atlas Mountains, a publication that speaks across languages and cultures through text, photographs and illustrations. This analysis is supported by interview excerpts that explore the artist/co-creator Abdelaziz Haounti's philosophical approach to visual communication, which draws on his use of sketches and drawings to transcend the limitations of language, through its detailed attention to the somatic and energetic life-worlds of equines, conveying details that elude the propositional form. The analysis highlights how socially engaged art can articulate challenging questions in ways that engage the audience and raise awareness of the welfare concerns of mules working in the tourism industry. It is argued that these contribute to a deepening, albeit more complicated, quality of relationship. We conclude that art that thus engages with multispecies societies involving humans and mules has an important role to play in addressing systems of oppression and exploitation in mountain tourism and any resulting loss of freedom.

## Introduction

Socially engaged art is a recognized field of practice in qualitative research and is increasingly being applied to addressing the complex health challenges we face in today's world (1). Although in the discipline of art history ‘socially engaged art' designates a specific genre of practice that is built upon community participation, often crucial collaborative aspects and involvement from the audience (2), the term, when understood literally as art that engages with societal issues, has also been applied in various disciplines transcending this disciplinary definition, including health (3, 4), social psychology (5), education (6–9), and anthropology (10, 11), typically to engage communities affectively with a view to effecting behavior change informed by social, environmental and multi-species justice (12–14). Given the interdisciplinary scope of this paper, as well as its place of publication, this paper will use this expanded sense of the term, beyond its strict art historical definition (2).

While in the field of art history, the capacity of art to address the ethical dimensions and dilemmas of animal exploitation has been an emerging strand over the last 20 years (15, 16), the use of art in developing guidance for human-animal coworking has received minimal attention in disciplines concerned with the subject of animal welfare, such as the veterinary sciences.

By taking the example of experimental art practices produced with the intention to help develop guidance for human-mule labor activity in Morocco, this paper seeks to contribute to the ongoing development of these practices. This work engages with the long-standing culture of shared labor between humans and equines in the country and the cultures of domination and exploitation that pass unquestioned (17), whilst also contributing to conversations on the role that art can play in a cross-disciplinary dialogue aiming to explore, understand, represent, and challenge human experiences, behaviors, assumptions, practices and cultures (18–21).

Where exploitation of the more-than-human world is concerned, socially engaged art practices can disrupt systems of oppression and stimulate transformational learning. This has particular relevance for relationships between equines and their humans, for the various ways equines are denied freedoms and for the potential co-creation of healthier relationships founded in a human desire to be a good listener and co-worker, and a friend, in a highly ambiguous sense. Friendship in this context remains an anthropocentric projection melding affective attachment, dependence and shared but unequal labor in the superstructure of the tourism industry. As such, an attitude of caution is encouraged when using this term to describe the interspecies relationships present in the Atlas Mountains, in recognition of the fact that these “friendships” are relations based on a transaction of care and labor, whilst remaining hierarchical. Such hierarchical relationships between humans and mules are by no means equitable, they can however be improved through attentional practice and practicing care. Art that is socially engaged can co-create new knowledges, helping us to reshape our understanding of relational care practices. Of course, the reciprocal relationships co-constituted by humans and mules in the mountains, as well as the care practices underpinning their shared work in the tourism industry are fundamentally unequal, as care inherently implies a power relation between caregiver and cared-for (22, 23). As previous research by Erdosi has shown, by focussing on art practices examining the extent, or rather, the non-extent of reciprocity between the animal and the human, and on the different ways in which anthropogenic attention manifests, perception can shift from animal labor to multispecies care, as art practices enable viewers to reacquaint themselves with taken-for-granted contexts of multispecies work and make them think about the care practices that interweave them (24). In this sense, we are emphasizing that art can be socially motivated and can seek to engage the audience in a range of ways with the intention of helping us better visualize the *betweenness*^1^ of our relationships (17). The audience can be imaginatively drawn into these liminal spaces when they are afforded opportunities and encouraged to attend to the marks and traces created by these relationships (20, 26).

Some marks (including tethering injuries, pack saddle injuries and bitting injuries) are injurious, reflecting the various ways the movements and freedoms of equids conflict with human desires and impositions (27). Others, such as the branding used to identify equids vaccinated against African Horse Sickness during the 1987–1991 outbreak (28), arguably reflect care efforts. The ability to notice, interpret and care about these marks can be developed and art can contribute significantly to this educational ambition. The imagining, production and sharing of visual materials thus constitutes an extended practice that seeks to move beyond their creation and distribution, rendering them educative and transformative. Their more-than-representational form (29) allows the relational complexities of everyday life, and in particular of muleteering practice, to be conveyed in “ways that words cannot capture and texts cannot convey” [(30), p.655]. As Rebecca Solnit [(31), p.58] writes “we in the West have been muddled by Plato's assertion that art is imitation and illusion; we believe that it is a realm apart, one whose impact on our world is limited, one in which we do not live.” Artists can help make the world for others “not only the material world but the world of ideas that rules over the material world, the dreams we dream and inhabit together” [(31), p.60]. When seeking to transform working equine welfare, reverie [Bachelard, (32)] and fabulation [Haraway (13)] helps us imagine other possibilities and other ways of living, working and dying together.

This paper sets out to provide an account of socially engaged practice for welfare and care, and demonstrate its potential to support advances in human-working mule justice^2^ in the Moroccan mountain tourism industry. Such advances acknowledge the many ways that both mules and their owners and handlers have been exploited by the tourism industry (33). This paper first provides a brief overview of the histories of art and literature that have concerned themselves with mule welfare. This allows us to recognize the many ways that human-equine relations have been represented in art and the extent to which care-ethics inform these practices. The paper then provides examples from a case study which has proven to be helpful in knowing working equids and, in particular, the mule. The analysis of this contemporary case study highlights how art and visual culture can constitute a tangible method to be implemented when fostering human-animal care practices in real-world cases, such as Morocco's tourism industry, and can even be privileged over written guidance. By bringing the case study into conversation with this historical overview, the research contributes to emerging strands of art historical literature on multispecies care (24, 34), to rapidly expanding scholarship in the Environmental Humanities that furthers the discourse on the inherent power dynamics of care, including its politics of violence and exclusion (24, 35–37), and to the One Health approach as it seeks to explore more equitable, reflexive and transdisciplinary approaches to health and welfare across the human and more-than-human realms (38). While building upon and contributing to these conversations, the paper's exploration of a tangible, real-world use of collaborative intermedia practices also helps us to understand, and ultimately, improve human-mule care and work relations in the specific context of mountain tourism. The conclusion brings these insights into conversation with equine welfare and One Health, seeking to identify where these may benefit from a more interdisciplinary approach, informed by an openness to all relevant disciplines and to the need to integrate different ways of knowing (39, 40).

The case study presented in this paper focuses on the work of two collaborating artists who have worked together on human-mule relations in Morocco. Cousquer's field photography has helped inform an extensive ethnographic study into the welfare of working pack mules in mountain tourism and how welfare can be improved through awareness-based systems change practices (25). Cousquer makes use of concerned photography (41–43) to direct attention to, and pose questions about, the quality and health of human-mule relationships. His work takes many forms but one of the consistent aims is to bring attentional and care practices into view. Abdelaziz Haounti's 2014 oil on canvas work *Hmirr l'bled* (the mule of the village) is an example of detailed observation of the many facets of a mule's movement, behavior and personality and their representation. This reflects Haounti's commitment to visual communication and how it can transcend the limitations of language, paying attention to the somatic and energetic life-worlds of equines. Together, they have contributed visually to the development of guidelines for pack animal welfare on expedition and to a publication that speaks across languages and cultures through text, photographs and illustrations (44).

As such, this paper contributes to the histories of tourism in the Atlas Mountains and puts forward the role of visual narratives in developing and tending to interspecies relationships in the region. In his book *Retour a Imlil* (2023), Moroccan anthropologist Mohamed Mahdi describes the rapid development of Moroccan-led local tourism in the 1990s [(45), p.128] and the presence of new roles linked to work with mules in tourism being a part of a diversification of income-generating activities. This so-called *pluriactivité* describes the emergence of work with mules alongside other forms of work linked to tourism, including guiding and cooking [(45), p.140]. Mahdi also underlines that the expansion of agriculture focussed on orchards and arboriculture and the economic expansion of tourism have paralleled the regression of other economic areas, such as breeding and grain-focussed agriculture (p.38). The labor of the mule is thus part of a broader ecosystem of labor implicating both humans and animals which underpins the layered and pluralistic nature of local tourism. The diversification of labor relations in the Atlas Mountains also emphasizes the fundamentally multispecies character of touristic and arboricultural labor in Morocco's mountainous regions.

In terms of the actual human-mule labor relations at stake, Andrea Giampiccoli's holistic perspective that integrates structural and personal attitudes to mule welfare is crucial when addressing human-equid relations in the region. His argument provides a key perspective on approaches to working mule welfare and is worth quoting at length here:

… especially in disadvantaged contexts, mule owners and handlers should not be misunderstood, and possibly automatically blamed, when they overuse the mule. For example, their overuse of the mule (or affected by other mismanagement actions such as saving from adequate veterinarian control) is for them a means of survival (however, and importantly, this does not justify the exploitation of the mule). In these contexts, it is necessary not to incriminate the owners and handlers of the mules but to understand them and recognize their living conditions and this also calls for the need to give them the necessary tools and resources which allow them to improve their livelihoods. It is necessary to facilitate to use (and not overuse) the mules in an efficient and balanced manner while safeguarding their overall welfare. This does not mean that mule owners and handlers should not carry their responsibility but this is to underline that all the involved stakeholders should be responsible for the mule's welfare—as such, responsibility needs to be balanced amongst all actors involved' [(46), p.8].

This perspective is also captured in work by Cousquer and Allison (47) which emphasizes shared responsibility across all actors operating within mountain tourism and subsequent work on the health and welfare of mules working on the Toubkal (48). While, as will be noted in the visual analysis of the guidebook / booklet that constitutes the case study of this paper, the case study publication occasionally falls short of the ideals outlined by Giampiccoli, it nevertheless puts forward an argument for the crucial role of visual culture in communicating issues of animal welfare between humans and mules in the region. Beyond this, it points to existing human-animal power relations in the Atlas Mountains and how they figure in local histories of the tourism industry.

Before turning to the main section of the paper, the positionality of the authors and their role in the writing process must be addressed and acknowledged. The paper represents an interdisciplinary and experimental collaboration between an animal geographer and veterinarian (Cousquer), who has worked in the High Atlas of Morocco since 2009 and spent 5 years teaching at the Mountain Guide School, and an art historian (Erdosi). The positionality of two white scholars working in a British academic context must be acknowledged, given the limitations stemming from cultural-political and geographical positions. The main case study to be analyzed contains photographs taken by Cousquer over an extended period (2008–2016) and represents one element of his long-standing collaborative practice with local artist Abdelaziz Haounti. Haounti was interviewed during the writing process and provided invaluable insight into his artistic process; as such, his voice is present as that of an artist, rather than a co-author. This decision stemmed partly from the art historical approach to develop insights based on interviews as primary sources, and also from the need to foreground Haounti's perspective as an artist, while also balancing the demands of academic labor that are necessitated by any type of collaborative work. The inclusion of his voice in the research process was crucial to the writing of this paper, as it provided an invaluable local perspective in terms of his artistic practice.

## Mules and working equines in literature and visual art

Mules and working equines have often figured in artists' works, raising important questions about how the work is to be interpreted and represents and contributes to human-equine relations. The French painter, Jacques Louis David (1748–1825), painted historical scenes from Napoleon's life and campaigns and became known for his paintings of imperial pretension (49). His painting of Napoleon's crossing of the Alps at the St Bernard Pass on May 20^th^ 1800, enroute to the Battle of Marengo, presents the first consul as “an unstoppable hero in the lineage of former conquerors of Italy, Hannibal and Charlemagne.” David's work is recognized as a form of historical fiction (50) and this painting was first and foremost propaganda, with Bonaparte asking David to portray him “calm, mounted on a fiery steed” (c*alme sur un cheval fougueux*). In reality, Napoleon had crossed the St Bernard on the back of a mule [(51), p.310] with three companions after his troops had passed. David's visual representation of the event underlines Patrick Vincent's point that the mule “does not enjoy pride of place in Western culture, and despite being stronger, more intelligent and agile than the horse, is rarely considered a noble creature nor the purveyor of sublime experience” [(52), p.214]. The mule's sure-footedness on mountain paths had no doubt informed the choice and is closer to the truth, despite its erasure from the picture.

Approximately 50 years later, the painter Paul Delaroche (1797–1856) was commissioned to produce a more accurate version of the crossing, which was completed in 1850. Adams (53) explores a deeper analysis of Delaroche's portrayal of Napoleon, arguing that Delaroche eschews the aggrandising qualities that characterize the first imperial era:

Delaroche portrays Napoleon as an alternately inspiring and tragic heroic who, when not inspiring admiration, inspires the bittersweet pangs of nostalgia. Though his Napoleon is sometimes idealized, as in Napoleon in his Study, he is at other times portrayed as accurately and unflinchingly as possible, as in Napoleon at Fontainebleau [(53), p.2].

Adams goes on to note that “Delaroche turned to printed images of the once-Emperor instead of officially commissioned portraits”; these included the work of printmakers and lithographers; works she characterizes as “inherently more democratic and populist” [(53), p.3]. Consideration of the portrayal of the equine is limited in these works, however, to their associations with the human protagonists the works purport to be about. The genre of history painting assumes a focus on the importance of the event depicted, as well as the figure that it is associated with, who in this case is Napoleon. As such, the genre implicitly underlines the subjugation of the animal subject, and even though the horse and the mule are represented in the works mentioned above, conceptually speaking, their labor remains invisible and is subject to perpetual erasure.

As Giampiccoli highlights in his paper:

[t]here exists an imbalance between the value of working animals in helping sustain human livelihoods and the value of their worth as assets. Working animals are fundamental for the livelihood of millions of people, especially in developing countries, however “working animals remain largely invisible in the eyes of millions, including amongst decision and policy makers, civil society, and donors, but also those who rely on them” [(54):XI] [(46), p.1].

In the case of the paintings in question, the role of the equids is only important in so far as their body elevates and moves the protagonist. As the painters' representational angles elevate Napoleon and the direction of light in the paintings draws the viewer's attention to the Emperor, the equids are relegated to decorative props that either contribute to the heroic tone of the painting (David) or are kept in the shadows, thus emphasizing the superiority of the human figure (Delaroche). This is of particular relevance to Morocco, given the way film director Rachid Bouchareb portrays the erasure of the significant work undertaken by North African mounted troops in the liberation of Sicily, Italy and France during the Second World War, in the 2006 film “Les Indigenes” (55, 56). This film was one of several used by Cousquer when teaching pack animal welfare at the Center de Formation aux Métiers de Montagne (2009–2014) to develop an appreciation of the shared history humans have had with mules when working or operating in harsh mountainous terrain, both on expedition and during wartime. The film presents the work of the mounted infantry and demonstrates the mobility of this unit, whilst also exploring the prejudice shown both to the North African French troops and mules and donkeys (57). A century and a half after the commission of the above-mentioned painting by Delaroche, the film allows for the representation of a more complicated multispecies history that provides a more detailed account of the role of the mule in key historical moments.

Fidelity to the truth, to the equine and to the relationships co-constructed by humans and equines is something writers and artists have continued to concern themselves with but it has taken time for concerns about welfare to emerge. In 2014, at the Hydra Mule and Donkey Conference, Jim Smyth presented a history of the mule and donkey in Irish art, highlighting the strong association between human poverty and deprivation and these working equids (58). Smyth's analysis of these paintings in the absence of written work on the donkey highlights a number of ways that artists have approached this question of this equine, including:

**i Romantic idealization and romantic nationalism**:
  ° Artists often presented donkeys as part of a rural arcadia, portraying Ireland as an idyllic, pastoral landscape. This was particularly prevalent among cultural nationalists who idealized the peasantry of the West as embodying traditional, uncorrupted Irish life. Donkeys were thus included in imagery that contributed to a narrative of a romanticized, pre-modern Ireland. Artists like Paul Henry depicted the West as a land of simple, happy peasants and their donkeys.
**ii Symbol of national identity**:
  ° The donkey was used to symbolize Ireland itself, serving multiple narratives. Donkeys were sometimes depicted as victims of exploitation, paralleling the Irish populace under colonial rule.
**iii Economic utility**:
  ° Visual representations often focused on the donkey's role in rural economies, particularly in the impoverished western areas, highlighting their practical use in agriculture and daily life.
**iv Cultural critique**:
  ° Some artists, like Micky Donnelly, critiqued the romanticized portrayals by disrupting traditional landscapes, offering a more critical view of Irish identity and history. This however, only emerged in the 1980s (58).

Smyth's analysis of the figure of the donkey in art is of pivotal importance in terms of its ecocritical framework that considers the contribution of their labor to agricultural life, thus designating the Irish agricultural landscape as a site of multispecies labor, and economic hardship in Ireland as shared by both humans and equids.

It is not just visiting writers who write and paint about working equines, seeking to render their stories and suffering available to a wider audience. Outside of Morocco, but well within the Mediterranean world within which mules played such a key role in rural life (59, 60), the Sicilian painter, Gianbecchina (1909–2001) looked at the peasant life-world of his native Sicily, poetically^3^ exploring the relationship man has with nature. This period of work extended from 1955 to 1981 and is referred to as “the age of the sickle”. Inevitably, mules and donkeys figure in these scenes, which capture many of the challenging aspects of peasant life shared by mules and humans as they work on the land (Figure 1). Ultimately, Gianbecchina's *Lungo Camino* echoes the critical perspectives put forward by Smyth; his painting represents the agricultural domain as a shared space of multispecies labor between the human and the donkey. While, of course, the sharing of the space is predicated upon inequality between the human worker and the animal worker, it nevertheless sheds light on agricultural labor as co-constituted and co-dependent upon (potentially non-consensual) human-animal collaboration. Choosing to depict the figure of the worker and the donkey as a dual portrait bestows a degree of dignity on both subjects, while also moving the representation of the agricultural world from angles of romanticisation that Smyth alluded to. This alternative way of seeing was described as lyrical realism: a turn or declination in art toward the non-ideological real.

**Figure 1:**
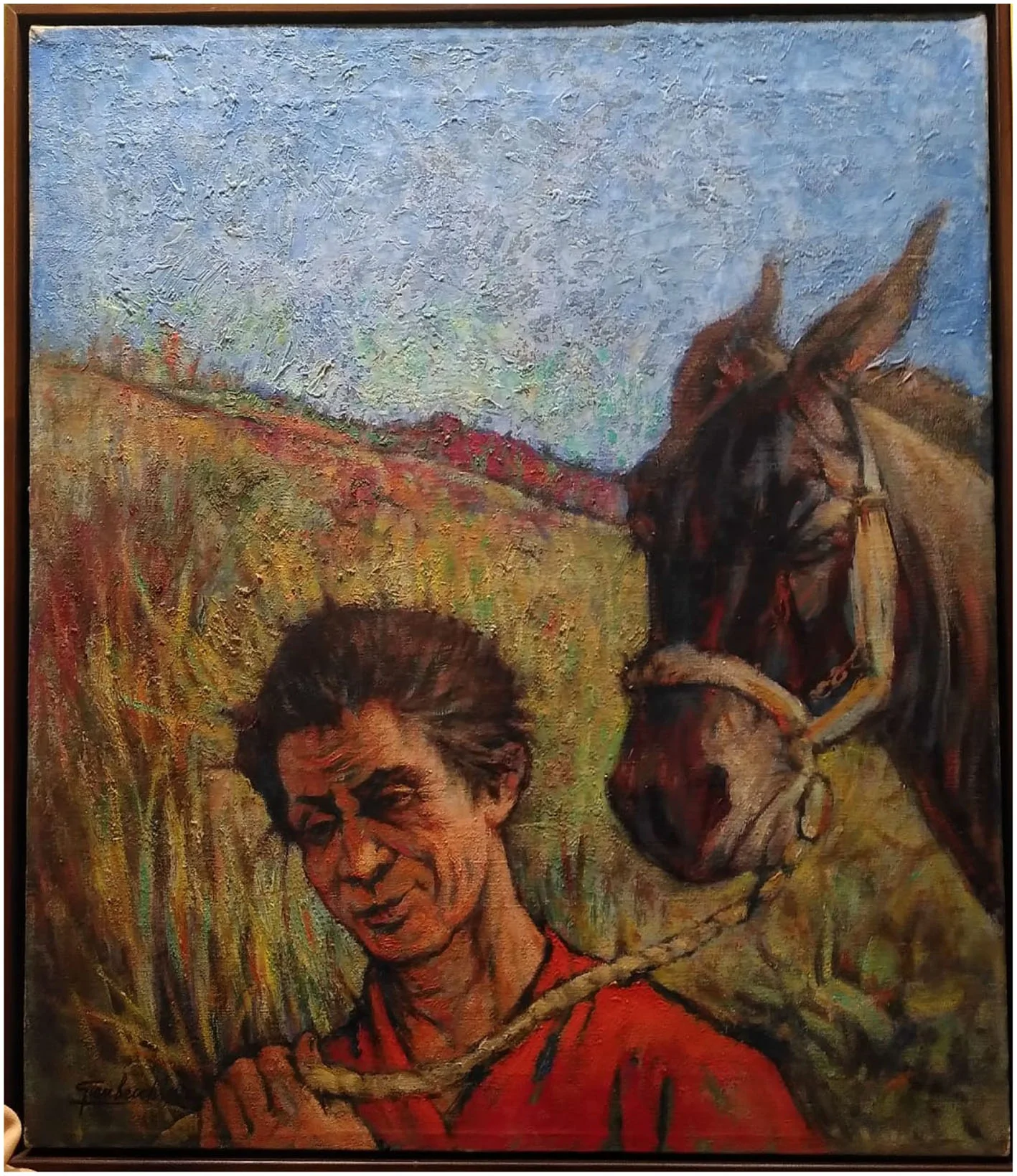
Lungo cammino—the man with the donkey walks for days, under the scorching sun, among spikes, vineyards, in the scorching sultriness of a sweltering day. He walks slowly and resigned with his hard workmate, that has the same resigned and exhausted expression. They come together toward another day of hard work. Image taken by the author at the Istituzione Gianbecchina [This cultural institution dedicated to the life and works of the renowned Sicilian painter Gianbecchina, is based in Gangi, Sicily (62)]. (Credit: Cousquer, with full permission of the Istituzione).

The working equid has been a recurring subject of literary works as well, but, as will be seen from this next section, the concern for the working mule in Morocco was often coupled with a colonial gaze toward the country and its human inhabitants. The emergence of socially concerned writers such as George Orwell drew attention to the plight and exploitation of workers and, arguably, of working animals. The dehumanizing effects of poverty and the many consequences of economic injustice are the primary or secondary themes of nearly all Orwell's work (63). Orwell was clearly concerned about the welfare of equids in general and, relating an incident in which he witnessed a boy beating a carthorse, writes:

It struck me that if only such animals became aware of their strength we should have no power over them, and that men exploit animals in much the same way as the rich exploit the proletariat. I proceeded to analyse Marx's theory from the animals' point of view. To them it was clear that the concept of a class struggle between humans was pure illusion, since whenever it was necessary to exploit animals, all humans united against them: the true struggle is between animals and humans [(64), p.88].

Orwell's account is remarkable in that it already alludes to the limitation of Marx's labor theory that has since been pointed out by numerous critical animals scholars, namely that his initial concept of labor does not extend to include that of the animal and regards it merely as an instrument of production in the hands of the human (65–67). Of course, Orwell's argument overly simplifies class struggle; speaking of it as simply representative of power dynamics between humans and animals is reductive, as it homogenizes the human species and disregards other sociocultural dynamics that play a part in class struggle, such as frameworks of gender or race, which intersecting politics have been examined by Bénedicte Boisseron (68) and Carol J. Adams (69), among others. Furthermore, as Wadiwel points out in *Animals and Capital*, considering animals as a class of their own can be beneficial to better grasp animals' role in capitalist production, allowing for an analytical framework that can address both human class struggle under capitalism as well as how animals are exploited under anthropocentric capitalistic order [(66), p.2].

Cole (70) in his detailed critique of Orwell's contradictions writes that Orwell is a “pursuer of political justice whose vision is fraught with a welter of contradictions so irreconcilable as to thwart his pursuit” (2017, p.35)^4^. In line with this, whilst not insensible to the suffering of animals, Orwell does not set out to tell their stories and move us to care for them. This is evidenced in his writing about the equids he observes when convalescing in Marrakech during the winter of 1939. As he writes:

I had not been 5 min on Moroccan soil before I noticed the overloading of the donkeys and was infuriated by it. There is no question that the donkeys are damnably treated. The Moroccan donkey is hardly bigger than a St Bernard dog, it carries a load which in the British army would be considered too much for a fifteen-hands mule, and very often its pack-saddle is not taken off its back for weeks together. But what is peculiarly pitiful is that it is the most willing creature on earth, it follows its master like a dog and does not need either bridle or halter. After a dozen years of devoted work it suddenly drops dead, whereupon its master tips it into the ditch and the village dogs have torn its guts out before it is cold [(71), p. 186].

It should be noted that this example is given by Orwell by way of contrast to the invisibility of the local people: “One could probably live here for years without noticing that for nine-tenths of the people the reality of life is an endless, back-breaking struggle to wring a little food out of an eroded soil.” [(71), p.184] and as a way of exploring the “culture of blindness … the very structures of seeing and not seeing and their consequences” [(72), p.163]. Nevertheless, the positionality of Orwell as a white Englishman furthers the problematic aspects of his point of view, whereby he chose to write about a Morocco that would only gain independence from France in 1956. His account of how animals are treated in Morocco could easily contribute to colonialist tropes about formerly colonized countries as “backwards or uncivilized,” which makes his point problematic on its own, but is made worse considering how it presents the mistreatment of animals as characteristic of the region, while animal exploitation has been prevalent in all countries across the world, including Orwell's home country of England.

A succession of other socially concerned writers visiting Morocco were struck both by the suffering of local people and local animals. Author Elias Canetti, in particular stands out for the voices and stories he brings to life in his record of a visit to Marrakesh. He starts *The Voices of Marrakesh* with an account of his encounters with *animals* decrying the suffering of the camels he sees being brought to the souks for butchering, of the deep impressions these scenes left upon him and of the gratitude he felt for the few illusory moments these creatures had been left alone (73). Later, in the same book, Canetti tells of a famished and wretched donkey:

Of all the city's miserable donkeys, this was the most pitiful. His bones stuck out, he was completely starved, his coat was worn off, and he was clearly no longer capable of bearing the least little burden. One wondered how his legs still held him up [(73), p.88].

This quote shows how Canetti demonstrated concern for the welfare of working equines when visiting Morocco, and gave voice to these creatures' stories, rendering them worthy of care and attention. However, it has to be pointed out that this does not absolve his work from the criticism that colonialist narratives permeate his text, as has been pointed out by Moroccan scholars Mohamed Tahar Es Siddiky and Yamina El Kirat El Allame. In terms of his use of language, for example, El Siddiky and El Kirat El Allame point out that ‘linguistic imperialism' infiltrates Canetti's *The Voices of Marrakesh*, whereby his “travel account perpetuates language hierarchy through mispresenting and marginalizing Moroccan local languages, showing them as ‘exotic', ‘inexpressive', and ‘inferior' compared to the colonizers' languages which were privileged in the novel.” As they continue, “linguistic imperialism in Canetti's travelogue is also expressed through the dehumanization of the natives' linguistic articulation, accusing them of being ‘incapable' of articulating correctly colonial languages. In addition to that, Canetti's novel maintains linguistic hegemony by privileging colonial languages such as French, English, and Spanish and legitimating their dominance over the local ones (Arabic and Amazigh languages)” [(74), p.11]. As such, while Canetti's writing clearly expresses an interspecies concern, this concern has to be read in a scholarly context that is sensitive to his book's fundamentally colonial undertones.

The pattern of expressing concern for the mule while furthering a colonial narrative is also present in the work of Gavin Maxwell, who had written an account of the social deprivation he had documented in Sicily (75), evidencing his deep concern for the exploited of this world. He demonstrated that this extended to the more-than-human when he found himself buying a white Arab stallion because he “could see no other way of saving this beautiful animal from the slow death by cruelty and neglect which awaited any creature that had outlived its usefulness in Africa” [(76), p.449]. As such, it can be reiterated that while Orwell, Canetti and Maxwell are clearly concerned with equine welfare, they also localize “cruelty and neglect... in Africa,” which, as has been mentioned in relation to Orwell, is problematic as it legitimizes and perpetuates the colonial gaze that perceives local culture as “uncivilized” and “neglectful,” and ultimately, as “the other.” Thus, they provide a deeply colonialist representation of the region that does not give voice to locals, but projects a Euro-centric, colonial perspective onto Morocco. Neglect and cruelty to animals beyond their usefulness are present in all regions of the world, and their scale reaches particular heights in the capitalist factory food production of the Global North. Furthermore, the accounts above fundamentally define the equid in terms of their suffering at the hands of the human; as Bénedicte Boisseron quotes Vicki Hearne in relation to animal rights literature, ‘the constant citation of Bentham, along with many other details, makes it as plain as anything can be that what interests some people is not the joy and intelligence and difficulty and difference of animals, but only their pain.' [(68), p.22] Ultimately, in the context of the human-mule relationship in Morocco, focusing exclusively on the suffering of the mule instead of the quality and politics of the relationship between the mule and the human forecloses a more direct engagement with how these relationships co-constitute multispecies labor on site, and how they might be ameliorated via the implementation of measures that are sensitive to the needs of the species involved. The overview above of how mules have been attended to by artists and writers provides helpful background to the exploration of the practices presented in this paper, which will now follow.

## Research approach

This paper aims to add nuance to cultural perceptions of human-mule relations outlined above, but is also an experiment in seeing how interdisciplinary work can evolve between the humanities and veterinary science. By calling for a deeper engagement with artistic-creative practices, it points to visuality's instrumental role in grasping the affective aspects of the labor that is undertaken in the Atlas Mountains. While the guidebook to be analyzed directs attention to the welfare issues at stake, through its distinct visual aspects it also shows that the labor undertaken by mules and muleteers and paid for by tourists is not emotionally neutral, but implicates hope, frustration and playfulness that can be captured by a visual approach. As such, the paper demonstrates how interdisciplinary research practices can address both affect and practicality when it comes to animal welfare. This section presents the approach taken to analyse the practices utilized by the case study. Visual work may concern itself with how we perceive the whole and understand the nature of reality. It is therefore important to start with an account of the methodological principles underpinning this work. Centring a visual practice in the analysis allows for a shift in the viewer's focus to the quality of the human-mule relationship in a veterinary context and presents them with a real-world case of how art can constitute a means to foster more equitable multispecies labor relations in the Atlas Mountains' tourism industry by way of visual, on-site guidance. While, arguably, written guidance can also be an effective way to provide guidance on the treatment of animals, the visual material in question, which juxtaposes drawing and photography, allows for a sense of immediacy when conveying its message about the working practices of the human and the equid in the mountains.

Attention, as Eviatar Zerubavel argues, is politically conditioned, for what one considers relevant or irrelevant is largely determined by social conventions [(77), p.8]. As he notes:

The selective nature of our attention is evident not only in the organization of our sensory experience but also in the remarkably similar organization of the way we think about as well as remember things. It is likewise evident in the cognitive organization of our moral concerns, as any given set of moral considerations effectively “goes out of focus” whenever a competing one “comes into focus” [(77), p.5].

Attention is also professionally conditioned (78) and there is therefore something expansive and potentially subversive in inviting an artist to help us see further and more imaginatively in a context that is heavily implicated in animal welfare concerns. The work of the photographer and the artist redirects attention to and focuses on specific aspects of the treatment of the mule and the shared practice of human-mule labor in tourism. The co-created guidebook project of Cousquer and Abdelaziz Haounti can also be instructive in regard to the ways in which the ethical implications of multispecies labor evolve on site, by drawing attention to expressions of care that can be developed between the two species. This point echoes and parallels the argument of previous doctoral research by Erdosi, which demonstrated how art practices can reconceptualise human-animal labor sites and reframe them as sites of interspecies care (24). The examination of interrelated practices of care and labor is transposed to a case study in the specific geographic region of the Atlas Mountains with its own specific interspecies implications. Furthermore, the present analysis and the visual narrative of the case study also aligns with a recent strand of broader interdisciplinary environmental humanities literature that concerns itself with attention and noticing as a way to foster a more attuned political engagement with the more-than-human environment (24, 79–82), and provides a situated account of what the politics of attention can entail for muleteers and equids in Morocco's mountain regions.

How we practice attending to the world, our “ways of seeing” (83) determines what we see and can attend to. This is well captured by John Dewey who wrote in his essay on the nature of the reflex arc that:

Upon analysis we find that we begin not with a sensory stimulus but with a sensori-motor coordination … and that in a certain sense it is the movement which is primary, and the sensation which is secondary, the movement of body, head and eye muscles determining the quality of what is experienced. In other words, the real beginning is with the act of seeing; it is looking, and not a sensation of light [(84), p.358–9].

We thus need to bear in mind that perception is an active rather than a passive process; or as Iain McGilchrist points out, “perception … is a profoundly interactive process”:

Perception appears on the face of it to be a passive process (in an age where the camera has become the false analogy for perception). Yet it is an active process in which we go to meet phenomena. There is widespread evidence that the motor and visual systems are intrinsically linked and mutually influence each other. Observations and perceptions affect movements, and movement affect observations and perceptions [(85), p.991].

These points by McGilchrist and Dewey do not touch on the political nature of attention and perception that Zerubavel referred to. As such, they need to be understood as part of a wider conversation, echoing Isabelle Stengers's point about ‘slow science,' which she makes to highlight the fact that scientific approaches should not lose sight of the political implications of their results (86). This therefore is part of the photographer's and the artist's craft, which must be understood as contributing to an unfolding conversation on multispecies politics rather than ending with the image captured and re-presented. As such, the manner in which the image is re-presented can engage the viewer in a much more inter-active process than we might at first assume. Methodologically, we might be prompted to let go of the habits of seeing that lead to fragmentation and to allow ourselves to see the flux and flow of reality, the whole in the parts [(87, 88)]. As Rebecca Solnit writes, “[t]he order that matters most is not spatial but temporal. Sometimes pictures convey this, but the habit of seeing in pictures encourages us to lose sight of the dance” [(89), p.189].

The methodological approach informing this work is rooted in process rather than analytical philosophy, a shift which still needs to be embraced in One Health (40) but one that is essential if we are to become true systems thinkers (90, 91) and appreciate that there are no objects of compassion because there are no objects, only subjects interrelating [(92) p.225]. The quality of interrelating can give rise to transformational change, where the radical interdependence of those who are relating is recognized and this gives rise to an opening-of- the-heart and an impulse to act to address the suffering (93, 94)^5^. This then is what it means to not lose sight of the dance. In one of his essays D.H. Lawrence wrote:

What is it that man sees when he looks at a horse? What is it that will never be put into words? For a man who sees, sees not as a camera does when it takes a snapshot, not even as a cine-camera, taking its succession of instantaneous snaps; but in a curious rolling flood of vision, in which the image itself seethes and rolls; and only the mind *picks out* certain factors which still *represent* the image seen [(95) p. 127–8].

The dance is thus a curious rolling flood but one that we can attend to through the different modes of attention afforded us by our two modes of attention that the psychiatrist and philosopher Iain McGilchrist argues is provided by each of our hemispheres. This choice is particularly relevant to practices of befriending because, as McGilchrist points out, what Lawrence describes perfectly is:

… the contrast between the seamless presence of experiential flow to the right hemisphere, and its representation in the left hemisphere, an inanimating takeover of the right hemisphere's animacy. [(85) p.958].

It is important to appreciate that the approach taken by the artists in this paper's case study may integrate and benefit from analytic insights (or snapshots) but it is firmly grounded in a surging iterative process of ongoingness, of relational processes unfolding, and of insights being integrated into practice. The booklet format in which Haounti's sketches were presented underpins this idea, as it allows for a process-based interpretation to unfold when looking at the drawings. This process recognizes that the images can help us as we shift our perspective from looking at the system to seeing ourselves in the system (88, 96). This is emergent strategy, a strategy that works with emergence and emergent complexity (97).

Transformation doesn't happen in a linear way, at least not one we can always track. It happens in cycles, convergences, explosions. If we release the framework of failure, we can realize that we are in iterative cycles, and we can keep asking ourselves—how do I learn from this? [(98), p.108].

This is the nature of learning and we have chosen to emphasize these onto-epistemological aspects of the work so that it is clear that we are situating socially engaged art practice in the context of the development of attentional learning practices, which, in the case study in question, are fostered by an educational booklet that supplements a host of other training initiatives. John Berger's oft-quoted line that “prophecy now involves geographical rather than historical projection; it is space not time that hides consequences from us” (1974, p.40) challenges us to consider what spaces and spacings prevent us from seeing and what sorts of spaces and practices might allow us to see more clearly. His reference to prophecy also provides us with an orientation toward and a focus on the future that can emerge through our choices as moral agents and communities. This is a further onto-epistemological orientation we wish to emphasize here because it underpins our creative methodology, and its emphasis on learning from the emerging future (88). Of such orientations, adrienne maree brown writes:

Creating more possibilities is my favorite aspect of emergent strategy—this is where we shape tomorrow toward abundance. Creating more possibilities counters the very foundational assumptions about strategy. The word strategy is *a* military term, which means *a* plan of action toward *a* goal. I want to emphasize the a's in that sentence—there is a practice for narrowing down, identifying one path forward, one strategy, one way, one agenda, one leader, one set of values, etc. Reducing the wild and wonderful world into one thing that we can grasp, handle, hold onto, and advance [(98), p.155–6].

Following on from brown's point, our creative methodology does not seek answers or singularity and we recognize that this may not deliver the clarity some readers may be looking for. It is, however, entirely consistent with the participatory collaborative co-creative ethos that lies at the heart of One Health, where equity across the sectors of human, animal and environmental health is emphasized as one of the five principles of One Health (39).

## Results

This section presents a selection of examples of the work undertaken by Cousquer with particular attention being paid to how the photographs were created. The paper will then turn to the analysis of the case study that collates Cousquer's photographs with Haounti's drawings in the framework of a single publication aimed at communities working with mules in the region.

### Cousquer's photographic practice

The images featuring in this booklet bridge what John Berger describes as “the abyss between the moment recorded and the moment of looking” [(99), p.91]^6^. Conscious of this abyss, Cousquer invokes Buber's invitation to read dialogically and imaginatively [(103) p.9], to enter into active give-and-take dialogue with these images as the reader develops their own awareness of the unique in-the-moment experience of what can emerge between mule and human when they interrelate.

In Berger's essay on appearances^7^, he presents an image of a horse and a man [(99), p.87] and notes that “the photograph offers irrefutable evidence that this man, this horse and this bridle existed. Yet it tells us nothing of the significance of their existence” (p. 88). He then continues:

Without a story, without an unfolding, there is no meaning. Facts, information, do not themselves constitute meaning. … when we give meaning to an event, that meaning is a response not to the known, but also to the unknown: Meaning and mystery are inseparable, and neither can exist without the passing of time. Certainty may be instantaneous; doubt requires duration; meaning is born of the two [(83), p. 91].

Elsewhere he asserts (p.98) that “photographs do not translate from appearances. They quote from them.” Through images and, subsequently through my interpretation of those images in words (both in the booklet and in other works), attempts were made to convey the welfare challenges by re-composing them, rather than by seeking to explain them [(61), p.117]. Two particular concerns emerged: tethering practices and overloading. An opportunity arose to produce banners for display in the village; for this, however, careful consideration was needed to ensure the final images used were not simple, static representations of a welfare concern but were instead dynamic and rich with questions and insights.

The image chosen for the tethering sign shows a mule straining against a thin rope tether (Figures 2A, B). The fractured hairs over the cannon bone are easy to see; this damage is relatively recent. Of much longer standing are the old rope burns in which the pigment producing cells within the hair follicles have been destroyed so that the hair grows back white. These marks, or traces, (20) can be seen as a series of white rings around the pastern and cannon bone. The mule is clearly pulling against the tether and is trying to reach some invisible but easily imagined need, perhaps forage or water, for these are often denied when the mules are “parked” up in the village stables, awaiting work while the young muleteers head off together to the village cafés. The image used for the overloading sign (Figure 2B) shows a heavily laden mule trotting down a steep slope. The muleteer is unladen and struggling to keep up, whilst in the far distance a tourist follows on behind. Both images carry questions about the practices the mule is caught up in, those of the mule handler, the tourist as well as the trekking agencies.

**Figure 2:**
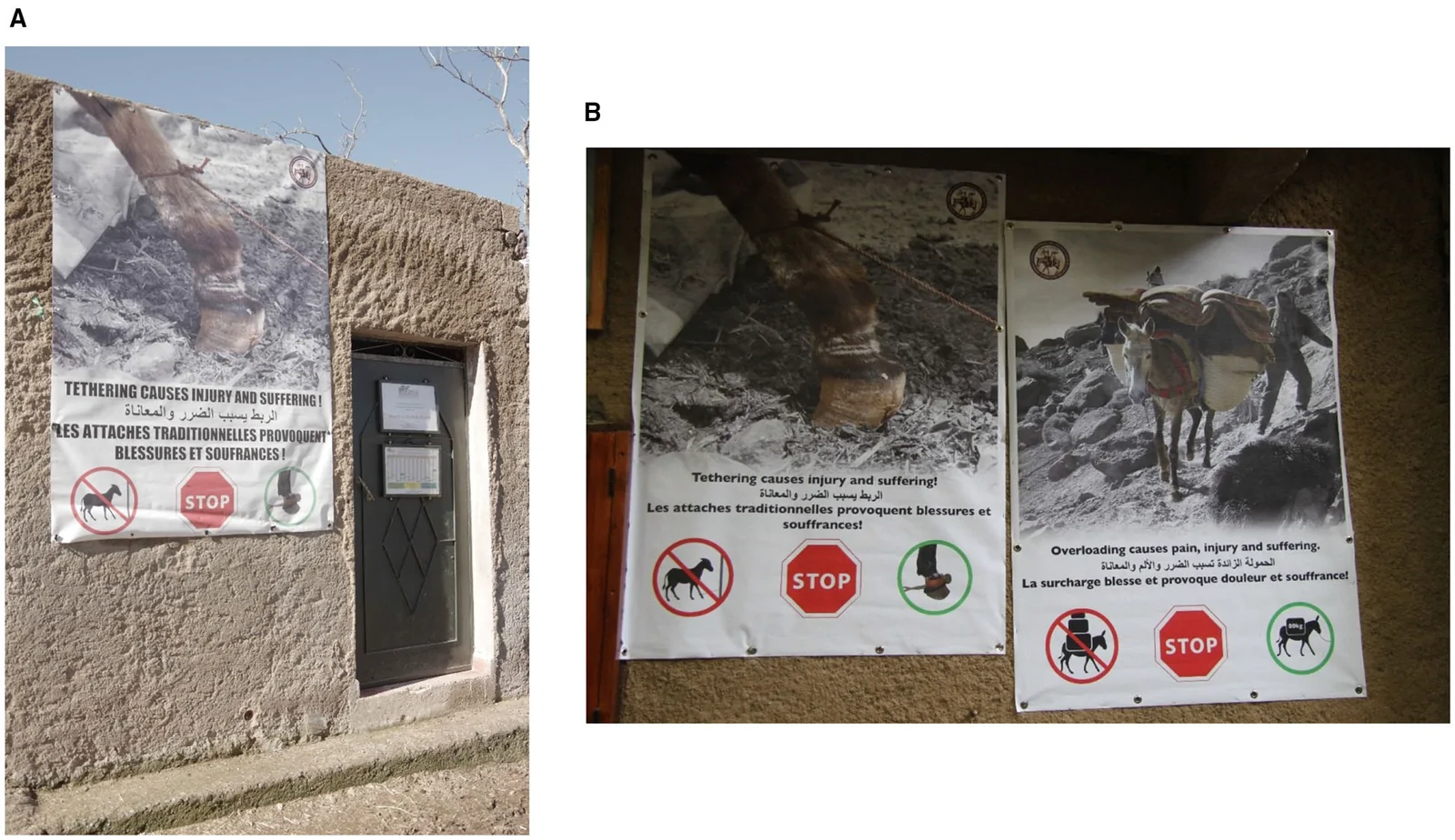
**(A)** The tethering banner is displayed in the mule parking at the foot of the village where mules were often left short-tethered with no access to forage or water. Grain is sometimes provided. The banner focuses on the tethered limb with the background rendered in monochrome. The caption emphasizes that tethering can cause both injury and suffering, especially where thin nylon ropes are used. **(B)** In the banner on the right, a mule on trek can be seen descending a steep slope between rocks, whilst heavily laden. This mule was supporting a team of two trekkers, their guide and muleteer, carrying all their equipment for a week-long trek. It is estimated that the load would have exceeded 80 kg given that the pack saddle typically weighs 20 kg.

The moments in time captured in these images had both a past (attitude, intentions and emotions) and imply a future (consequences). These were not leant but evidenced^8^; these photographs were therefore meaningful [(83), p.91] for they allow for an understanding of how the past feeds into the present moment, giving rise to the future that unfolded after the photograph was taken. As such, the relationships between mules and humans can become a focus of attention and of future concern in terms of interspecies welfare. In particular it becomes possible to attend to the deeper relational elements of the system: the social structures and institutions that drive feedback and the underlying values, goals and worldviews of actors. At this deepest systems level, it becomes possible to see the mindset (s) out of which the system arises and imagine ways of transcending that paradigm [(91), p.4].

### Analysis of the main case study: a guidebook aimed at local guides in the tourism industry to help them improve human-mule labor relations and conditions

Combining text in English, French and Arabic, as well as photography by Glen Cousquer and cartoon-like drawings by Abdelaziz Haounti, the guidebook/booklet in question (44) provides written and visual guidance on the treatment and welfare of mules working in the tourism industry in Morocco's Atlas Mountains. The publication was created in collaboration with travel associations and donkey sanctuaries, thus demonstrating the importance of the issue in the region and the industry as well as showcasing a collaborative effort between photographers, artists, commercial stakeholders and animal welfare organizations.

The guidebook opens with an introduction, but as its narrative progresses, text becomes sparse and the photographic and graphic elements of the publication take center stage and become more pronounced. These pictorial aspects of the guide shed light on the entwined and shared nature of human and mule labor, be it manual or metabolic (Figures 3–6). Ultimately, the images highlight the human-animal power relations pertinent to the context of the Atlas Mountains; the mules can be *better* cared for in the mountains, but this does not mean that they will stop being subjected to the needs and wants of tourists, as well as their owners and caretakers.

**Figure 3:**
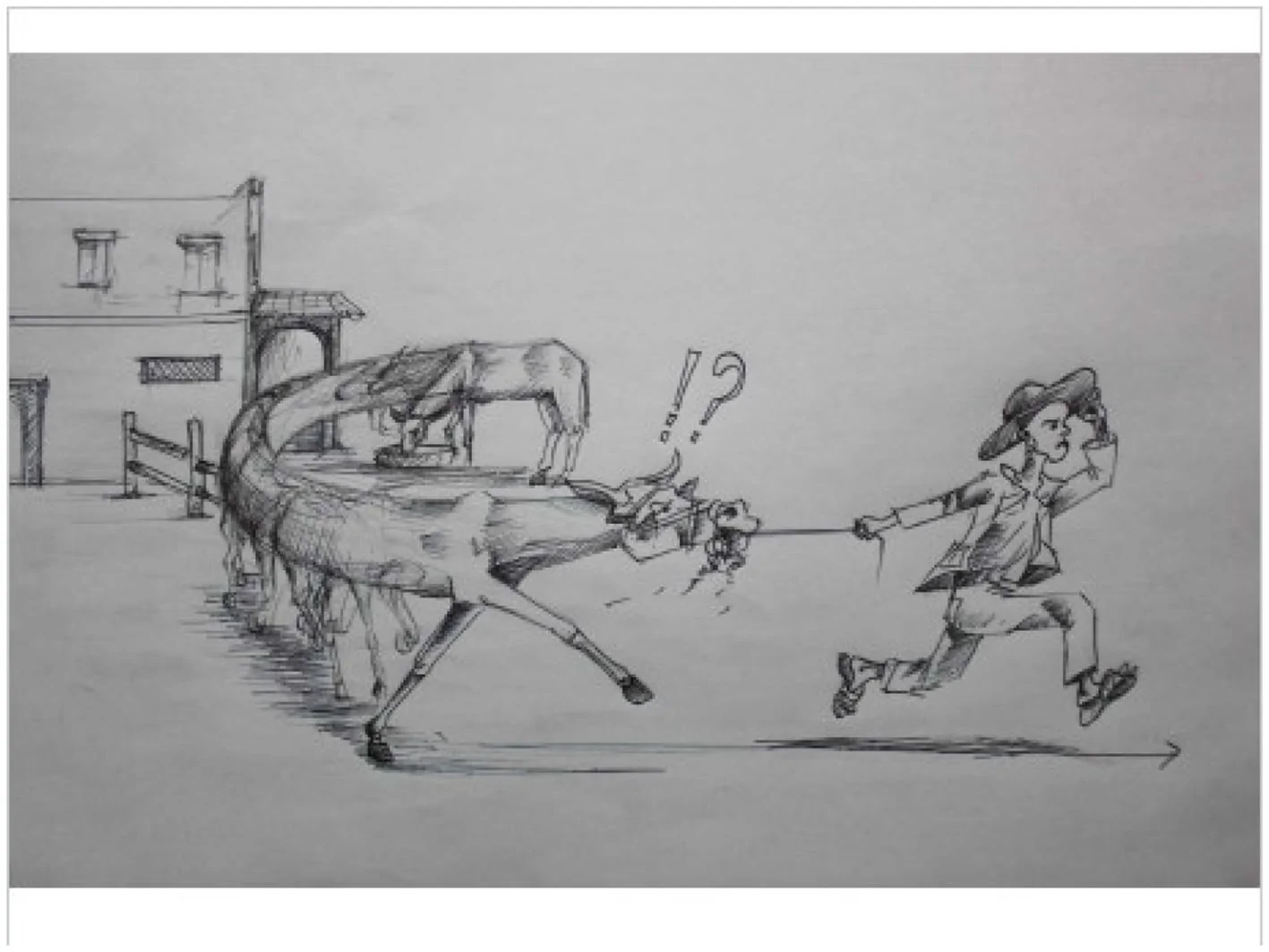
This image captures the brutality of the traditional bit, which can allow mules to be forced to move forwards in order to avoid the pain produced by the bit's action. These effects were captured in video form which may have inspired this drawing. The image also captures the interrupted meal and the forced departure. Courtesy of Abdelaziz Haounti.

**Figure 4:**
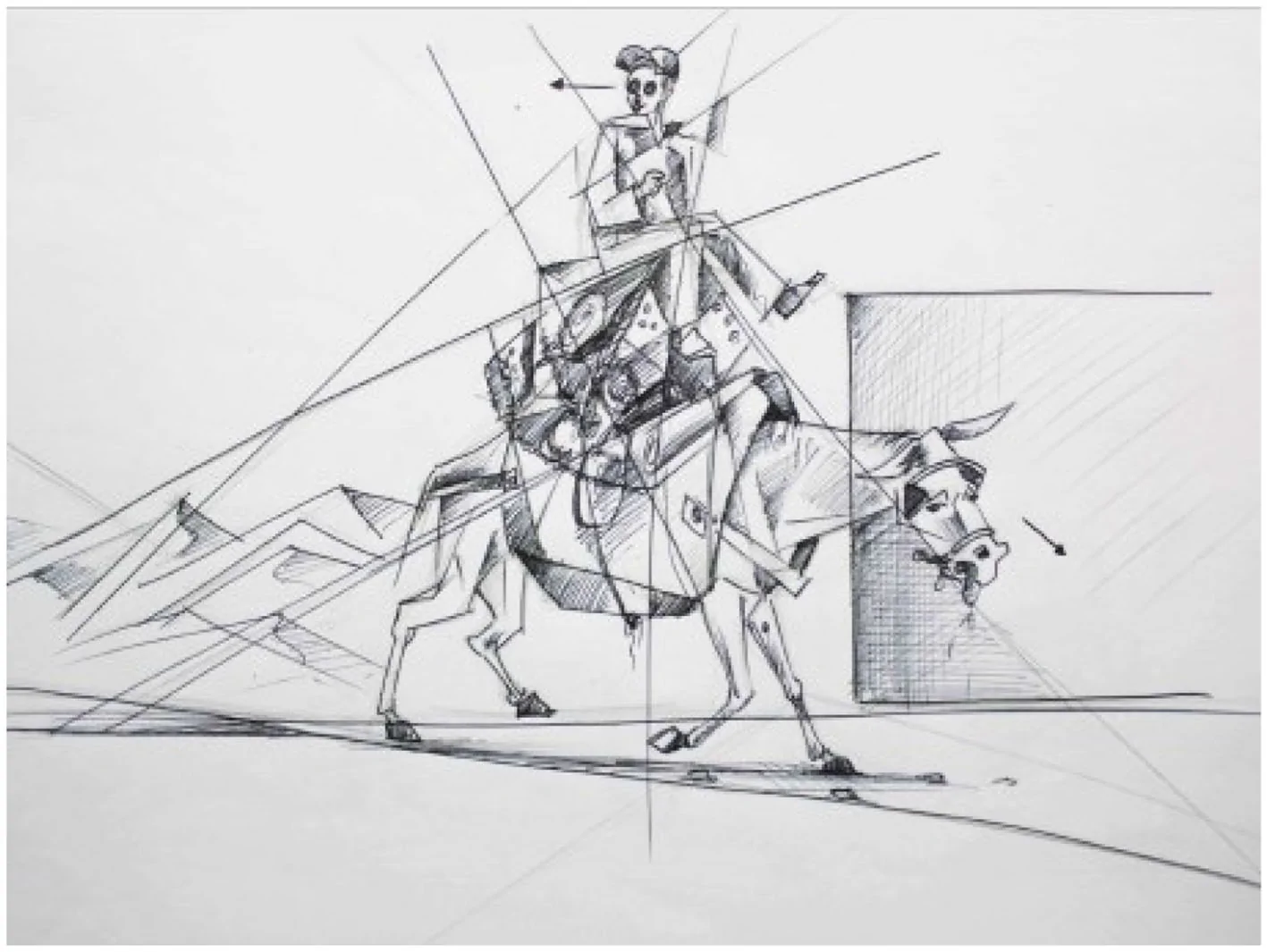
This image captures an extreme example of overloading often seen on the trail where muleteers choose to add themselves to their mules' load rather than walk. It also speaks to the inattention to the mule and the mule's experiences and welfare. Manual and metabolic labor. Courtesy of Abdelaziz Haounti.

**Figure 5:**
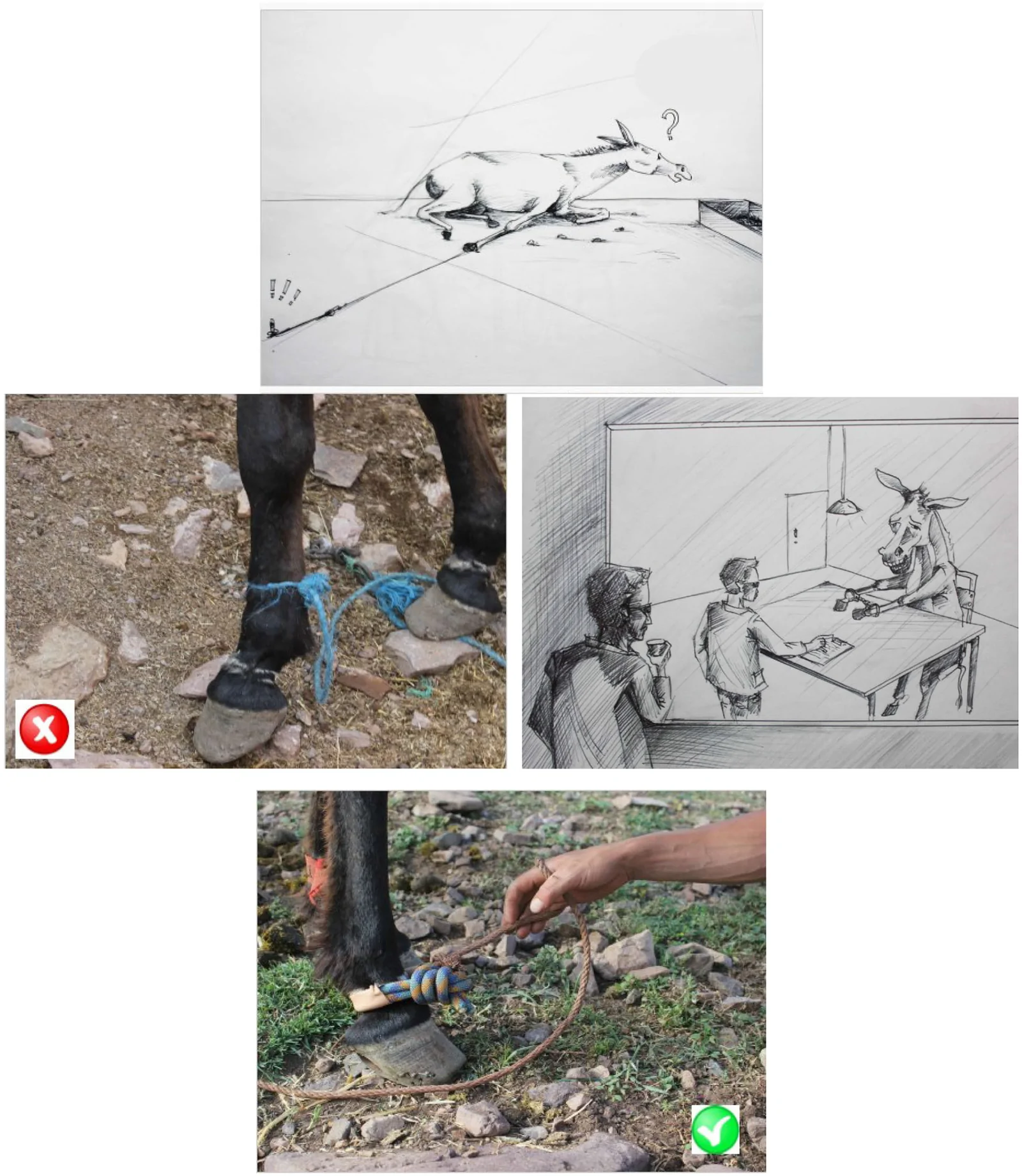
**(A-D)** This set of images capture the injuries, pain and suffering that tethering can cause and one alternative technique that makes use of retired climbing ropes to widen the contact surface area and reduce pressure. Drawings: Courtesy of Abdelaziz Haounti.

**Figure 6:**
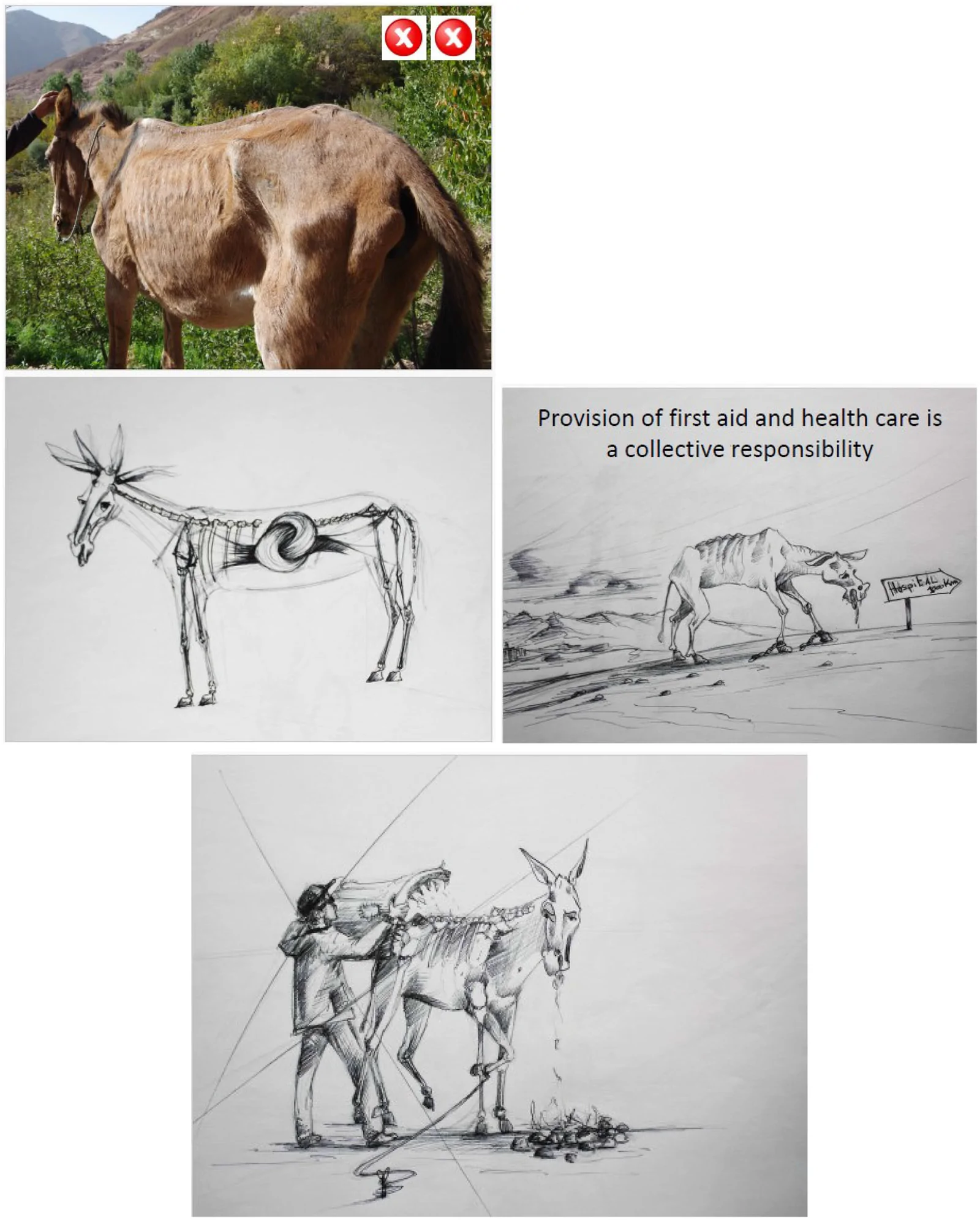
**(A-D)** These images capture the importance of removing the pack saddle and examining the mule carefully to establish their body score and the state of their back which is often subject to saddle sores in thin mules, where the pack saddle does not protect boney prominences. The images also highlight the need for a healthy diet and for both first aid and health care. The latter needs to be considered by trekking agencies who typically have viewed guides as employees but not muleteers and their mules. Drawings: Courtesy of Abdelaziz Haounti.

In the introduction, the call for better care sounds almost utopian: ‘[w]hat is required of man is that he stop and take time to reflect on his actions toward mules and donkeys. He must ask himself and be able to recognize if his behavior toward this animal does not reduce them to the status of an OBJECT or MACHINE which man simply uses to accomplish his tasks and works.' In fact, a mule working in tourism will always be *used* precisely for their physical labor and as such, they will never cease to be used as a means of production. Care is a relation of power, and care and violence often go hand in hand, as has been pointed out in both in feminist care theory and in animal studies (22–24, 35, 37). However, this does not mean that care is futile; as Puig de la Bellacasa argues:

Rather than give up on care because it is enlisted in purposes we deplore, we need to have its meanings debated, unpacked and re-enacted in an implicated way that responds to this present. Care is too important to give it up to the reductions of hegemonic ethics. Thinking in the world involves acknowledging our own involvements in perpetuating dominant values rather than retreating to the sheltered position of an enlightened outsider who knows better' [(36), p.10].

Exemplifying the above, the guidebook does not shy away from showing the results of violence inflicted upon the animals. However, instead of using them as devices of shock, a tactic often employed in mainstream animal activist media (104, 105), their function becomes to present a counterpoint to the advisable course of action vis-a-vis the mule, thus taking on an educative role regarding human-mule work in tourism. Their use in the booklet serves the purpose of instruction and transformation, rather than mere shock.

The images (both photographic and drawn) also clearly hint at the collaborative aspects of the labor involved in tourism. As Haounti underlined in the interview the authors of this paper conducted with him, he perceives the relationship between the working mule and the working human as a mirror; he has found that the behaviors of the animal and the human often mirror each other, thus underpinning the deep connection that can be fostered in the context of tourism (Haounti, 2026). While the mules working in the mountain environments around the village of Imlil are often seasonal workers^9^, and thus the depth of their relationship with human muleteers is constricted by their labor's temporal limits, the booklet's instructive illustrations nevertheless point toward the potential to deepen interspecies connections without ever idealizing them.

In the booklet, the photographs often portray the issues resulting from the potential maltreatment of the mules, such as tethering wounds. In contrast to these portrayals, the depictions of the drawings offer more ambiguity, whereby the high stakes of the human-mule working relationship are coupled with a playful, sketch-like visual language. As Haounti expressed, the rationale behind this visuality, beyond simply being intuitively drawn to the medium, was stemming from his intention to tackle a serious eco-social issue with a sense of humor that would hopefully help the viewer remember more easily the issues at stake (Haounti, 2026). This attitude aligns with Corey Lee Wrenn's research on the effectiveness of vegan activist media that outlines that repeatedly showcasing the most tragic details of animal abuse is a strategy that comes with demonstrably mixed results [(104), p.385]. As seriousness and playfulness meld on the pages of the booklet, the images express an underlying trust toward their viewer, which appeals to their judgement that can hold hopefulness, light-heartedness and empathy together, hopefully resulting in more considerate actions toward the working mule.

Fostering a more equitable relationship with the working mule also echoes the societal ethics of the Indigenous people of the region, as the artist, Haounti, pointed out when explaining the importance of fostering humility toward the mule through his visual practice:

I wanted to pull here from the Amazigh history. In terms of its relation to nature, it teaches you how be humble. It is a sort of respect and also a kind of surrender. One of the key things that really fascinates me is that for Amazigh people there are no hierarchies. It doesn't matter how much money you have or wealth, but what matters instead in relationships is how humble you are toward nature and your value in the community. People treat each other with respect according to what they offer to the community and how they treat nature and animals and children. It's not about how much money you have or what your status is (Haounti, 2026).

As such, the artistic intention behind the drawings has a cross-temporal aspect, whereby societal traditions and instructions in terms of the treatment of the animals are implicitly co-represented on the pages today. It is important to not idealize the human-mule relationship, as it forever remains a power relation between the human and the mule, and is fundamentally transactional both between the mule and the muleteer, as well as the guide and the tourist (47); this transaction involves both an embodied transaction implicating human and mule bodies, as well as financial transactions between tourists and local people. While this double-layered transactionality persists even in the framework of the booklet, the very existence of the guidebook, and its use distribution at training workshops, underlines existing hopes for more equitable relations and a trust in visuality to communicate this. However, it is important to note that the labor of the mule is seasonal, and there is often a yearly turnover in the specific mules who work in mountain tourism. This temporality underlines certain limitations of the human-mule relationship, whereby the depth of the relationship between the human and the equid is limited by this seasonal change in the animal workforce.

### Issues

There are a few points in the publication where the content could be better contextualized or evaluated, which, when addressed, would allow for future developments. These occasionally would necessitate crucial corrections in terms of intercultural framings, as well as interhuman power dynamics. Two such instances are outlined below, the minor but crucial modification of which could significantly alter the intercultural impact that the publication may convey.

(1) The booklet opens with a contextual text explaining the importance of caretaking in light of ‘man's' tendency to make the mule suffer and neglect their wellbeing (44). Such a preface is certainly crucial and complements the imagery found in the booklet, but it has to be pointed out that the text in its current form contains problematic elements in terms of its characterization of local attitudes; ‘[a]nimals suffer because of man and the way in which man abuses his power. Donkeys and mules are among the most exploited and ill-treated animal. This is especially the case in poor, landlocked and traditional countries, regions lacking communication and exchange and where ignorance can persist.' The assertion above does not put forward contextualization and historic engagement with the deep-seated impacts of decades of colonization impacting local economies, and singles out countries as ‘traditional' and ‘ignorant,' which furthers a simplified and racialised narrative about the region as uncivilized; as it has been said in relation to Orwell's writing, ignorance, neglect or suffering inflicted on animals based on normative ethics and traditions are rampant in many other parts of the world. This includes countries of the Global North that dominate the animal farming industry, thus subjecting animals to a capitalist-industrial exploitation that is normalized, generally accepted and profitable. The excerpt, importantly, centers the wellbeing of the animal, which is one of the primary goals of the publication. However, this could be developed in future editions in a way that refrains from attributing qualities, such as ignorance, to entire regions, which may result in a sense of ‘othering' and perpetuates generalizing and racialised tropes. Considering that the guidebook is used in training local guides and is also available at guesthouses welcoming tourists, it would be crucially important to use language that does not perpetuate colonialist stereotypes.
(2) There are further elements coming across as problematic, for example, in the page illustrating the dangers of overloading the animals; in this particular instance, a woman and a child are shown from the back, and a text “> 80kg!!” alerts the reader to the issue at stake (Figure 7). Overloading the mule is certainly a risk and can be detrimental to the health of the animal, but this important message could have been presented differently, perhaps with an emphasis on the need to consider the carrying capacity of the mule, which emphasizes respect for the mule, without appearing to comment on a person's body size and weight.

**Figure 7:**
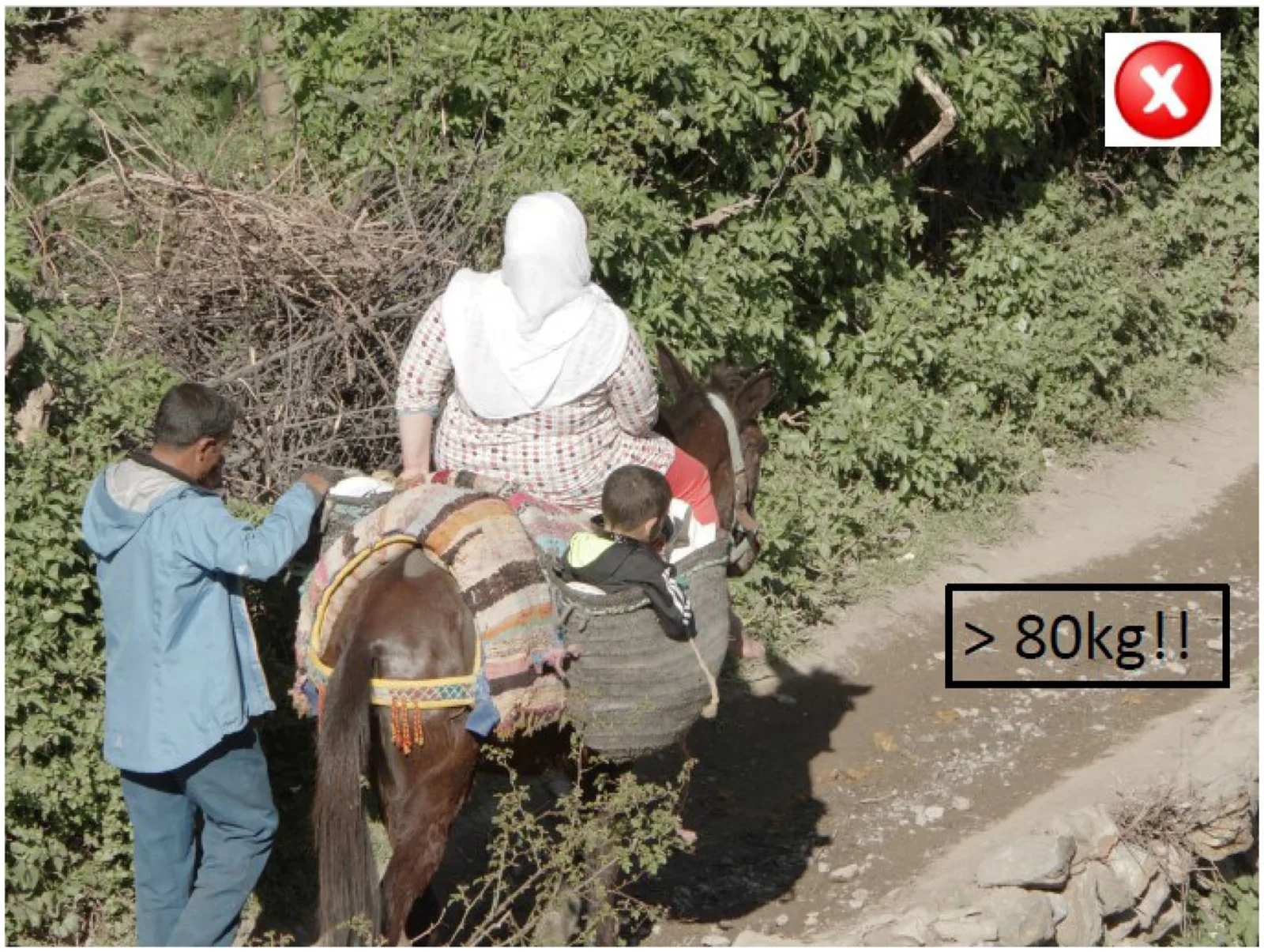
Weight comment.

The examples above should be amended in future editions of the booklet. The premise of the guidebook and the key concept to utilize artist-drawn pictures that accentuate the affective aspects of labor and human-mule relationships puts forward a real-world use case of how art can be used for the betterment of animal welfare in the Atlas Mountains, and remedying the issues stemming from cultural stereotyping and derogatory wording in terms of body image could ultimately also result in a more sensitive and stronger visual and textual work. This would also be preferable to foster a sense of the environment as one where human and animal laborers co-constitute the work environment, while not denying the existence of the fundamental power imbalances which are present in such a space.

## Conclusion

This paper has reviewed the literature on socially engaged practice for human-mule welfare, before presenting an analysis of the work of a photographer and an artist who collaborated on the development of guidelines for pack animal welfare in Morocco's Atlas Mountains. This publication offers a combination of imagery that aims to direct the viewer's attention to objective welfare concerns and encourage the viewer to appreciate the possibility and preferability of alternatives, thereby seeking to raise awareness, elicit concern and foster actionable steps toward more equitable human-mule relationships. These are complemented by more subjective representations of working mules, depicted in drawings and sketches. This blending of different photographic and drawn media allows for the acknowledgment of the different modalities of reading and visual interpretation that the viewer will likely employ; the guidebook posits that actionability and practicality can and should meld with affect when turning toward human-mule labor relations.

Locally, the material was shared with local ground handlers and their teams of guides and muleteers, as well as with several professional or industry bodies, including the Expedition Providers Association (EPA) and the Adventure Travel Trade Association (ATTA), the international industry body that seeks to promote guiding standards across adventure travel. This has meant the reach has extended beyond Morocco to the countries where these international organizations operate (such as with guides working with mules in the Andes). Further research will be necessary to fully grasp the impact it has had on muleteering practices both in Morocco and across the world. Given the international scope of its dissemination, the booklet is an example of locally significant efforts reaching a global scale, thus fostering not only interspecies, but also interhuman connections via the use of visual media.

The analysis of the booklet presented here highlights how socially engaged art can articulate challenging questions in ways that engage the audience and raise awareness of the welfare concerns of mules working in the tourism industry. It is argued that these contribute to a deepening, albeit more complicated, quality of relationship. In the same way that Duguma et al. (106) highlight the value of role-play as a way of learning to empathize with mules and donkeys, this work recognizes that creative visual work can engage the audience imaginatively and experientially, helping them relate to the lived experiences of working equines. We conclude that art that thus engages with multispecies societies involving humans and mules has an important role to play in addressing systems of oppression and exploitation in mountain tourism and any resulting loss of freedom. This provides equine welfare with ways to move beyond the transmission of explicit knowledge where objective facts are emphasized and into a relational field where the objective gives way to the subjective and inter-subjective (where ways of relating between subjects can evolve).

The role of artists in questioning power relations and abuses of power, especially in countries which were themselves colonized and oppressed is significant. Vogl (107) notes that contemporary Moroccan artists “consider art as a springboard for research and knowledge” (p.235), noting art's ability to contribute to their country's development and to “offer the public new ways of seeing and appreciating the many facets of contemporary art” (p.236). If this is true of art, it is likely to also be true of the social, environmental and multispecies justice issues that artists choose to focus on and bring into the consciousness of their public. At the recent *Why Look at Animals?* exhibition at the National Museum of Contemporary Art in Athens, nine works by the Moroccan artist Mostafa Saifi Rahmouni exposed the ritualistic and consumerist aspects of the violence inflicted on animals and the extent to which our relationships are inseparable from power and exploitation systems [(108, 109), p.54]. This example illustrates the important role played by contemporary Moroccan artists in evolving “ways of seeing” (107) and of caring.

This leaves us with important questions about how a more radically interdisciplinary One Health approach that integrates a wider range of relevant disciplines can contribute to the advancement of health and welfare (40). We do not pretend that these can be answered, for they remain to be explored. What is clear is that there is growing recognition of the need for collaboration between artists and those typically working within the health and welfare disciplines such that we can see more deeply into the current realities and creative possibilities that a more open collaborative multispecies ethic can open to.

What are the implications for working equine welfare? The work presented here was informed by the Hand model of animal welfare developed by the Donkey Sanctuary for use in its international work (27, 106, 110), the simplicity of which can help demystify animal welfare (111). The Hand looks at (i) relationships and behavior, (ii) body condition score, (iii) wounds, (iv) lameness and (v) other signs of ill health [(106) p.6], whilst also considering the whole life of the equine. This recognizes that “a holistic take on welfare considers every aspect including some that often remain invisible such as the provision of opportunities for friendship, social interaction and natural behavior, rest and recovery” [(27), p.15]. The knuckles of the hand represent the interconnections, reminding us “to think about local cultures, attitudes and working practices, which sit at the interface between the life of the mule (the palm) and the indicators of welfare (the fingers) [(27), p.15]. It is this sense of storied interconnectedness that visual practices can help us to engage with, explore, question and reimagine. As Solnit emphasizes [(31), p.106] “empathy is the capacity to feel what you do not literally feel” and this can be extended to the nonhuman world, further enlarging our sense of self (p.115) as relational beings who can find themselves co-working with equids. This is particularly easy where we have shared experiences and a shared capacity to empathize (112), a connection that artists and socially-engaged art are well placed to articulate.

## Data Availability

The original contributions presented in the study are included in the article. Further inquiries can be directed to the corresponding author.

## Footnotes

1 The between space is a liminal territory that arises in relationship. Its conceptualisation has been developed by Martin Buber and arises through a turning from an *I-It* relationship into one characterized by the *I-Thou* [Cousquer, 2023; (25)].

2 A form of multi-species justice (12) that recognizes that both mules and their owners/handlers have been exploited by the tourism industry.

3 The poetics of the work can be understood epistemologically as working from the whole. In the words of M.C. Richards “Poetry brings images of wholeness into consciousness, recombining what the rational principle has severed. But the severing has been meaningful, for by it we have discovered the parts and their architecture. When poetic cognition redelivers us into unity, it is the richer for our having now the powers of discriminated data and an aware self. A community of awakened men” [(61), p.91].

4 He continues: “On the one hand, he refuses to disavow our animality, rooting his vision of democratic socialism in a biopolitical awareness of our bodily capacities and vulnerabilities. On the other hand, he refuses to fully avow our animality either, instead taking recourse to traditional humanist tropes of ennoblement. This may be interpreted, on the one hand, as a defensive gesture, an attempt to repudiate the dehumanizing mobilization of the category of the animal by the totalitarian menace he devoted his political life to combating. On the other hand, however, Orwell himself evidences a propensity to animalize and thus inferiorise those whose relative humanity he wishes to gauge, thus partaking of the totalitarian ethos he so vigorously disparages. And finally, on the one hand, he reveals himself to be aware of our exploitation of animals to the extent that the specter of animal suffering at our hands actually motivates the composition of one of his most-read works. And yet, on the other hand, this awareness seems not to affect his essential stance toward animals, as he ridicules vegetarians as espousing an inhuman antisociality while ignoring the possibility that concern for animal welfare might constitute a plausible motive for their dietary choices” [(70), p.350].

5 This approach is fundamental to awareness-based systems change, which recognizes the primacy of consciousness as the source from which systems arise.

6 John Berger's collaboration with the photographer Jean Mohr is also evidenced in their publication “A Seventh Man” that explores the exploitation of migrant workers (100–102).

7 In (100) *Another way of telling: A possible theory of photography. London: Bloomsbury*.

8 The story is part of the photograph not imposed upon it or leant to it. The photographs thus provide evidence of welfare concerns including loss of freedoms.

9 Some mules may be bought to work during the tourist season and then sold on again but many families will keep their mules for many years and build significant relationships with them. Mules kept over winter will work less during these colder months and will spend more time indoors. At the same time, there will be days when they are allowed to roam freely above the villages.
